# Devastating disease can cause increased breeding effort and success that improves population resilience

**DOI:** 10.1098/rsob.240385

**Published:** 2025-05-28

**Authors:** Laura A. Brannelly, Danielle K. Wallace, Alexander S. Wendt, Quinn Higgs, Siyu Zhang, Marcus A. Hough, Ke Diao, Eleyne Ferguson, Lee Berger, Lee F. Skerratt

**Affiliations:** ^1^ Melbourne Veterinary School, Faculty of Science, University of Melbourne, Werribee, Victoria, Australia

**Keywords:** chytridiomycosis, amphibian reproduction, wildlife disease, conservation, breeding display, parental analysis

## Abstract

Novel and invasive diseases are a key threat to wildlife. The disease chytridiomycosis has had devastating global impacts, but some amphibian species can persist and even rebound after severe declines. Understanding how these species persist is critical to discovering management techniques for supporting declining species. Here, we explored the impacts of disease on reproduction in the threatened *Litoria verreauxii alpina*, investigating its effect on reproductive effort and success using a combination of laboratory-based clinical trials and field sampling through capture–mark–recapture surveys. We found that male frogs are increasing various facets of their breeding effort resulting in increased offspring. Infected male frogs (i) increased vocal sac coloration, (ii) increased sperm quality, and (iii) fathered more egg masses than uninfected males. Our research demonstrates that frogs can counteract high disease-caused mortality through enhanced breeding effort, which could lead to population persistence. Mitigation for wildlife diseases often aims to directly reduce mortality, such as through increasing host resistance or decreasing environmental suitability for the pathogen. Our work indicates that reproductive output is also important and should be considered when protecting our precious amphibians.

**Figure:**
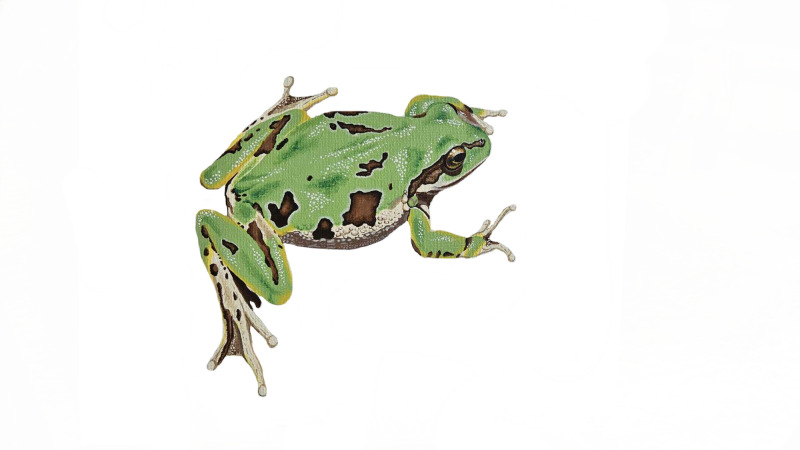

## 1. Introduction

Introduced pathogens are an emerging concern for wildlife around the world, and the impacts of these pathogens are likely to be underestimated [1]. With increased globalization, the pathogens too are able to move and spread across the globe and wreak havoc on ecological communities [2]. Some recent examples of introduced pathogens devastating wildlife are the H7 high pathogenicity avian influenza that infects seabirds across the world [3] and white-nose syndrome in microbats in North America [4]. Perhaps the most well-known and well-studied example of a wildlife disease causing massive population declines and even extinctions is chytridiomycosis in amphibians [5]. However, despite disease-related declines in >500 species and extinction of >90 species globally [5], many amphibian species and populations are persisting despite endemic infection and disease-related mortality. To best safeguard at-risk amphibian species, we need to understand how some species persist with high disease-caused mortality and why others continue to decline. Unravelling the mechanisms of population persistence in the face of endemic disease will help conserve amphibian populations but also serve as a case study to identify the ways in which species can persist with introduced pathogens more broadly.

Since the discovery of chytridiomycosis in 1998 [6], much research has been devoted to understanding the impacts of this disease on amphibians globally. A primary focus has been on understanding immune mechanisms and environmental co-factors that could be exploited for mitigation [5,7]. But in many cases where amphibian populations are surviving with endemic disease, there is no evidence of these factors contributing to amphibian persistence [8]. For these species, increased reproduction via ‘terminal investment’ might explain their continued survival [9–12].

The terminal investment hypothesis refers to the trade-off between investing in one large but final reproductive event versus investing in survival and future breeding [13]. Rather than expending energy into fighting disease at the expense of breeding, which is commonly reported in the biological literature [14–17], animals infected with disease can respond by increasing their reproduction (i.e. fecundity or breeding compensation). This increased reproductive effort in lieu of investing in self-preservation can enable their genes to be passed to the next generation and can ensure that the population persists to the next breeding opportunity. The terminal investment hypothesis has support in a wide variety of animal species, where increases in reproduction following exposure to a pathogen or antigen have been demonstrated in mealworms, house sparrows, limpets and Tasmanian devils [18–21], as well as in frogs [9,10,22]. For species that are not investing in increased immunity or changing their behaviour or habitat choice to help reduce pathogen burden, terminal investment appears to be a mechanism that enables population persistence [8].

Recent research [8–10] indicates that increased reproduction might be critical to understanding how amphibians persist despite endemic disease. However, mechanisms for improving reproduction and exploiting sexual selection are poorly understood within a disease context. The interconnections are further complicated because hormones important for reproduction like testosterone and corticosterone can reduce immune responses, or more fecund males can be exposed to and maintain infections more often than less fecund males [23–25]. Teasing apart reproductive effort and success in response to disease/infection needs to be conducted using both field studies (to understand the system within the appropriate ecological framework) and with targeted randomized-control laboratory studies (to understand the specific impacts that disease has on specific reproductive parameters in a controlled environment).

**Figure 1.**
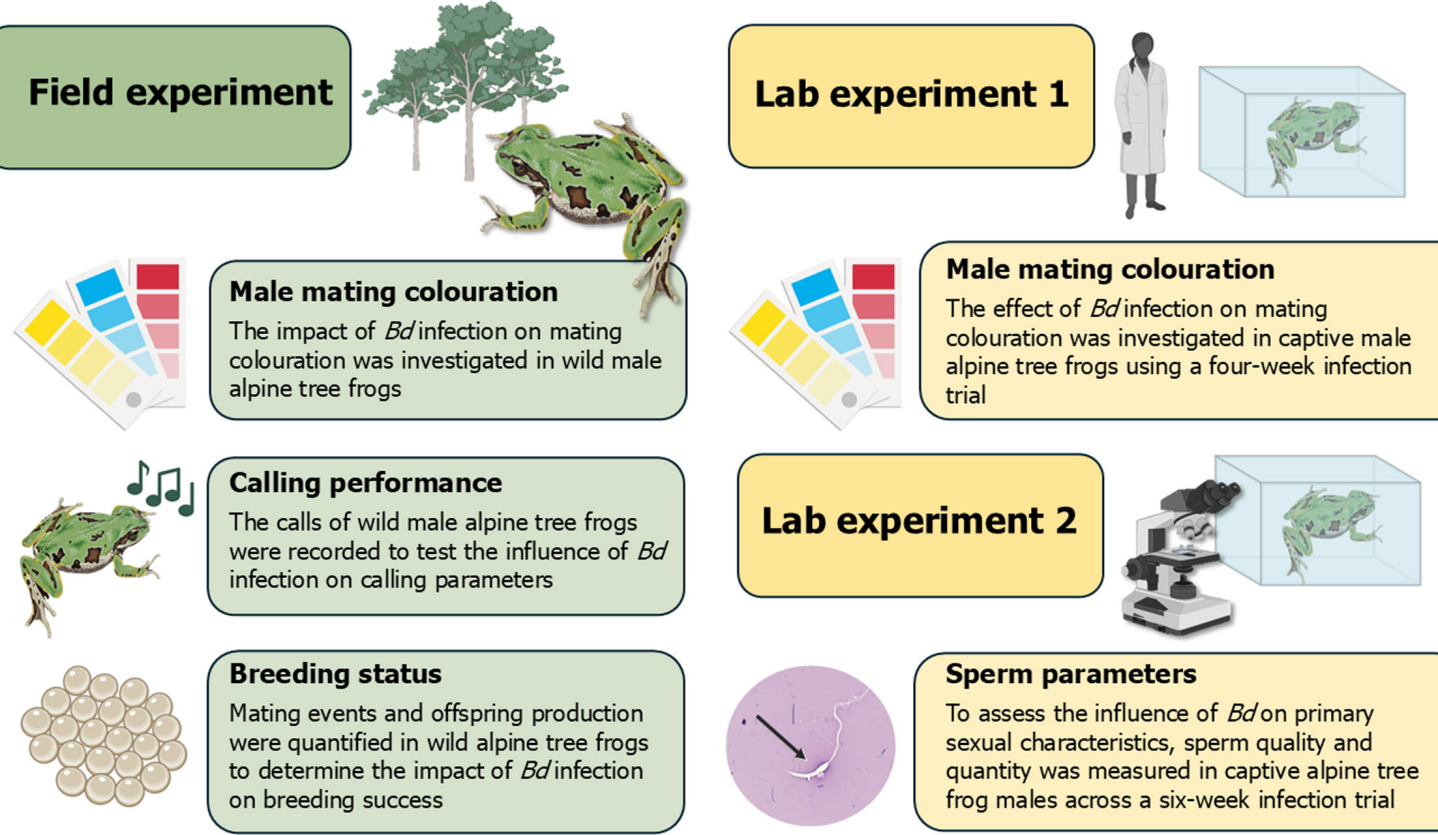
A visual overview and summary of the individual laboratory and the field experimental components of the study.

In this multifaceted study, we explored how disease impacts primary and secondary sexual characteristics, as well as breeding effort and success, using the endangered and highly susceptible alpine tree frog, *Litoria verreauxii alpina*, as a model. This species has declined from more than 80% of its range due to chytridiomycosis, yet persists at some sites despite high annual mortality, owing to high annual recruitment [8–10,26–28] (see electronic supplementary material, text S1). This frog-disease model can help us predict species’ responses to infection and is important for prioritizing management actions for threatened species. Here, we conducted a series of laboratory and field experiments (figure 1) to explore the impacts of disease on male reproduction. We examined secondary sexual characteristics like male calling displays and male mating coloration, as well as primary sexual characteristics like sperm quality and quantity. We then assessed if the observed increase in male reproductive effort led to functional change by quantifying mating events and offspring production using paternity analysis and instances of amplexus.

## 2. Methods

### 2.1. Secondary sexual characteristics

#### 2.1.1. Clinical infection trial

Adult (1-year-old) male *L. v. alpina* were randomly (see electronic supplementary material, text S1) allocated to each treatment group (*n* = 30, 15 infected and 15 control). Frogs were housed individually in terraria (34 cm × 20 cm × 15 cm) on a gravel and moss substrate at 16−19°C 12:12 h light:dark cycle during the austral spring, which is their natural breeding season. Terraria were flushed daily with carbon-filtered water and were fed crickets dusted with vitamin powder (Repashy) twice per week for the duration of the experiment. Power analysis indicates that a sample size of 15 per treatment group allows us to detect a 3.1% difference (proportional change) in colour measures among treatment groups at *α* = 0.05, power = 0.8.

Males were inoculated with a known virulent strain of *B. dendrobatidis*, the pathogenic agent of chytridiomycosis (electronic supplementary material, text S1): 5 × 10^5^ zoospores if exposed, and mock-infected if unexposed (electronic supplementary material, text S1). Frogs were swabbed for *B. dendrobatidis* (see §2.3), weighed, measured and analysed for breeding coloration weekly. The snout-to-vent length (SVL) to the nearest 0.1 mm was measured using dial callipers, and each frog was weighed to the nearest 0.01 g using digital laboratory scales. Colour readings were taken using a portable spectrophotometer (see §2.1.2), beginning in week 0, before inoculation. The experiment ended after four weeks when some animals began to show clinical signs of chytridiomycosis (e.g. anorexia, irregular skin slough, splayed leg posture).

#### 2.1.2. Skin colour methodology

Colour readings were taken using a portable spectrophotometer (OceanOptics USB2000+), probe (OceanOptics R400-UV-VIS) and light source (OceanOptics PX-2 Pulsed Xenon light source). Three repeat colour readings were taken from the dorsum, throat and venter of each frog. The probe was disinfected with ethanol after every individual. See electronic supplementary material, text S1, for setting and output details.

### 2.2. Field trial for colour and calling characteristics

#### 2.2.1. Field sampling

Our surveys were conducted during the austral spring over five sampling times between 19 October and 18 November 2018 (site information in electronic supplementary material, text S1). We sampled a total of 45 male *L. v. alpina* for call recordings, colour readings and infection. Power analysis indicates that a sample size of 21 per infection group (infected or uninfected) allows us to detect a 2.6% difference (proportional change) in colour measures among groups at *α* = 0.05, power = 0.8.

Once a calling male was located, we used a Marantz Professional hand-held solid-state recorder (PMD6661 MK11, InMusic, Japan) and a directional microphone (Rode Microphones, Sydney, Australia) to record calls of male frogs (electronic supplementary material, text S1). The recorder was set up 1 m away from the calling animal and the field team retreated to at least 10 m away from the frog once the recording began to minimize disturbance to the animal. We recorded the calling male for a maximum of 20 min. Prior to capture, we measured the temperature of the frog and substrate using a dual laser infrared thermometer (RS PRO 8861 ± 1°C). We captured frogs using clean, gloved hands. We swabbed, weighed, and measured the frogs.

We collected colour readings for male *L. v. alpina* in the field using the same method as outlined in the laboratory methods above (and electronic supplementary material, text S1), except that in the field we only took throat coloration measurements.

#### 2.2.2. Call analysis

We obtained call and infection data for 39 male *L. v. alpina*. From the call recordings, we chose five complete calls per male for analysis. We analysed the calls using the sound analysis software Raven Pro (Cornell Lab of Ornithology, Bioacoustics Research Program). See electronic supplementary material, text S1, for call parameter details. Power analysis indicates that a sample size of 10 per group (infected and uninfected) allows us to detect a 3.8% difference (proportional change) in call parameter measures among groups at *α* = 0.05, power = 0.8.

### 2.3. *Batrachochytrium dendrobatidis* testing

To collect a sample for *B. dendrobatidis* from each frog, a dry sterile rayon swab (MW-113, Medical Wire and Equipment, Wiltshire, UK) was rolled five times along each of: side, venter, thigh, each hand and foot. The swab was stored at −20°C until processing (approx. three months). Skin swab samples were extracted using PrepMan Ultra (Thermo Fisher Scientific) (electronic supplementary material, text S1). We used qPCR (Rotogene, Qiagen) to amplify and quantify the *B. dendrobatidis* DNA in each sample following standard procedure with modifications [29–31] (electronic supplementary material, text S1). Any sample was determined to be *B. dendrobatidis* positive if the reaction well was amplified, representing at least 2 ITS DNA copies detected in the sample. Samples below this threshold were coded as 0 DNA copies [29].

### 2.4. Primary sexual characteristics

#### 2.4.1. Clinical laboratory experiment on sperm quality and quantity

Animals were housed individually following the same husbandry procedures as clarified in §2.1.1. We selected individual males (*n* = 58) from our *L. v. alpina* captive colony when they were 1 year old and sexually mature in the austral spring. Animals were allocated to the two groups: exposed to the fungal pathogen (*n* = 29) or unexposed control animals (*n* = 29) (electronic supplementary material, text S1). We followed the same exposure protocol as used above (§2.1.1; electronic supplementary material, text S1); however, animals in the infected group were exposed to fewer zoospores (3 × 10^5^ zoospores in 5 ml Milli-Q^®^ water). We exposed these animals to a lower infectious dose to try to extend the experimental duration to six weeks before clinical signs were present.

Each week, animals were swabbed for *B. dendrobatidis* (following the procedures in §2.3), weighed and measured. Every two weeks, a subset of the animals was induced for spermic urine collection. Animals were only induced once during the duration of the experiment. We successfully collected samples from all 29 control animals (9 on week 2, 10 on weeks 4 and 6). Two exposed animals had either cleared infection or were never infected at the time of sample collection; therefore, a total of 27 individual samples were collected for the infected animals (10 on week 2, 8 on week 4 and 9 on week 6). Some samples produced few sperm; therefore, not all analyses were conducted on all samples, for example, viability analysis was able to be conducted on a total of 48 individuals (see electronic supplementary material, table S3). Power analysis indicates that a sample size of 24 per treatment group allows us to detect a 2.45% difference (proportional change) in call parameter measures among treatment groups at *α* = 0.05, power = 0.8.

We used exogenous hormones to stimulate and increase the release of sperm into the spermatic urine, using Chorulon (a purified hCG) [32]. Using a 27 g needle, 120 IU of hCG was injected into the peritoneal cavity from the ventral side. One hour later, we collected spermatic urine using a fire-polished glass Drummond microcapillary tube (20 μl) that was inserted into the cloaca. We determined cell concentration using a haemocytometer, sperm motility using 30 s videos, sperm viability using eosin–nigrosin staining procedure [33] and sperm morphology via the eosin–nigrosin stained slides (electronic supplementary material, text S1).

### 2.5. Mating success and offspring production

#### 2.5.1. Data collection

Frogs were captured from the field via a capture–mark–recapture survey, where animals were surveyed weekly for six weeks (electronic supplementary material, text S1). Individuals were sexed and morphometric data were taken, including SVL (mm) and mass (g), skin swabs for *B. dendrobatidis* infection load, toe clips and pictures for identification for recaptures (electronic supplementary material, text S1). At each recapture throughout the survey, morphometric data and skin swabs were collected and individuals were identified via photograph. At the time of capture, if a male was actively in amplexus with a female, we separated the two animals, but noted which female the male was actively engaging with (electronic supplementary material, text S1). We identified pairs to be in amplexus if both the male had a tight grip and the female was not actively attempting to escape the male’s grip. Power analysis indicates that a sample size of 145 infected in the field and 145 uninfected allows us to detect a 12% difference (proportional change) in breeding success among infection groups at *α* = 0.05, power = 0.8.

Newly laid *L. v. alpina* egg masses were identified weekly, and four eggs were collected with a disposable 2 ml pipette from different areas on the outside edge of the egg mass (to minimize egg mass disturbance). The individual eggs were stored in 100% ethanol until processing for genotyping.

#### 2.5.2. Parentage analysis

To assign parentage to egg masses, all adult toe clips, as well as three eggs per egg mass (165 total eggs), were genotyped. Tissue samples and eggs were sent to Diversity Array’s Technology, which utilizes the genotype technique DArTseq™ (electronic supplementary material, text S1), and we used the program Colony to determine the parentage of the egg masses [34–39].

#### 2.5.3. Animal ethics and permits

All work was conducted under approval of the University of Melbourne’s Animal Ethics Committee (applications 1814537 and 10267), and Wildlife Act 1975 Research Authorization permit numbers 10008829 and 10010126.

#### 2.5.4. Statistical analyses

All statistical analyses were conducted in R within the RStudio interface [40,41]. We assessed model assumptions for linear models (LM) and linear mixed effects (LME) models to ensure that no model assumptions (normality, homoscedasticity, independence, and linearity) were violated via different visualization plots of the residuals. We conducted null hypothesis testing, where for the field studies, possible fixed effects variables that were included in these model comparisons were size of the frog (log_10_(mass, g)), frog temperature (°C), air temperature (°C), humidity, site and date of survey. Infection status (whether they were positive for *B. dendrobatidis* infection or not; unexposed and infected for laboratory trials, and infected and uninfected for field trials) was always included in the model. For laboratory studies, the possible fixed effects included mass. Mixed effects models were conducted where clutch was included as a random effect, although if the variance was equal to zero, clutch was removed from the model. Treatment (infected or unexposed), week and the interaction of treatment and week were always fixed effects. For both laboratory and field trials, if infection status/treatment was a significant predictor variable, then infection load was assessed as a fixed effect. To explore whether load was a fixed effect, the data were subset by only animals that were infected (returned a positive qPCR result for *B. dendrobatidis* infection), and then infection load (log_10_ DNA copies of the ITS region) was included as a fixed effect in the model. When we conducted LME models where each individual was repeatedly measured (repeatedly over weeks, or multiple readings taken within a sample), then the individual was a random effect in the models. To determine if the possible fixed effects (e.g. possible covariates) improved the model fit, we conducted model comparisons using Akaike’s information criterion (AIC) and chose the model with the lowest AIC as the best fit model. Models within two AIC were considered equal, and we then chose the simplest model. We calculated the effect size via Cohen’s *d*, and Tukey’s *post hoc* tests were performed where appropriate. We used an alpha threshold value for statistical significance of 0.05 unless otherwise stated in electronic supplementary material, text S1. See electronic supplementary material, text S1 and tables S1–S4, for information on the multiple specific models performed for each study.

## 3. Results

### 3.1. Infected males increased their throat breeding display coloration

**Figure 2.**
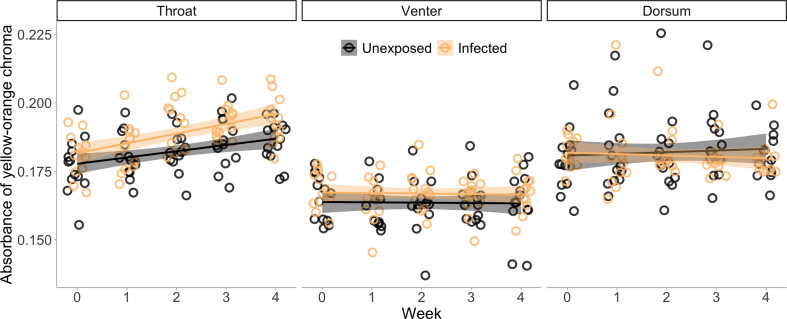
The change in skin colour over the four week infection experiment. There was a significant effect of infection and week on throat coloration, where infected animals overall had more yellow-orange coloration in the throat, and their coloration increased over time. Conversely, in the venter and in the dorsum, there was no statistically significant effect of infection on skin coloration. In each panel, each point represents a single individual each week (12 individuals that were infected and 13 individuals that were unexposed to *B. dendrobatidis*). These single points represent an average of three colour readings per frog per week per skin location. The lines are the smoothed conditional means for each of the unexposed animals and the infected animals. The shading around the lines represents 95% confidence intervals.

In a controlled experiment, we randomly infected animals with *B. dendrobatidis* (15 infected, 15 unexposed) and took spectrophotometer readings of their throat to record colour parameters. All exposed animals became infected with *B. dendrobatidis*. We found that yellow-orange chroma (570−620 nm) in the throat patch was 3.8% higher in infected *L. v. alpina* (*d* = 0.73) across weeks 1−4 than in uninfected animals overall after exposure to the pathogen. And there was a significant interactive effect of week and treatment, where over the infection experiment, infected animals increased in yellow-orange chroma, while unexposed animals did not (LME: treatment, χ^2^ = 4.418, *p* < 0.001; week, χ^2^ = 158.079, *p* < 0.001; treatment × week, χ^2^ = 7.828, *p* < 0.001; figure 2; electronic supplementary material, table S1). Yellow-orange chroma was directly affected by the fungal burden; as infection load increased so too did the yellow-orange chroma in the throat (LME: load, χ^2^ = 30.031, *p* < 0.001; electronic supplementary material, table S1, figure S1a). Prior to inoculation, there was no difference in yellow-orange chroma between the infected and unexposed treatment groups (LME: χ^2^ = 0.999, *p* = 0.314; electronic supplementary material, table S1).

In contrast, laboratory-induced infection had a significant negative effect on ultraviolet (UV) chroma (300−400 nm) in the throat patch, where infected animals (weeks 1−4) had 14.8% less UV chroma (*d* = 0.71) than healthy frogs (LME: treatment, χ^2^ = 5.012, *p* = 0.025; electronic supplementary material, table S1). We also found a significant negative effect of infection load on UV chroma in the throat, where the UV chroma decreased with increased infection burden (LME: load, χ^2^ = 5.891, *p* = 0.015; electronic supplementary material, figure S1b). Prior to inoculation, there was no difference in UV chroma between the infected and unexposed treatment groups (LME: χ^2^ = 2.319, *p* = 0.128; electronic supplementary material, table S1).

To confirm that the changes in throat coloration were due to breeding display rather than skin pathology caused by *B. dendrobatidis* infection, we tested the animals’ dorsum and venter colorations throughout the experiment. We found that there was no difference in venter or dorsum coloration (yellow-orange chroma, 570−620 nm; UV, 300−400 nm; or brightness, 300−700 nm) over the infection experiment between infected and unexposed individuals (see electronic supplementary material, table S1, for all model results; figure 2).

**Figure 3.**
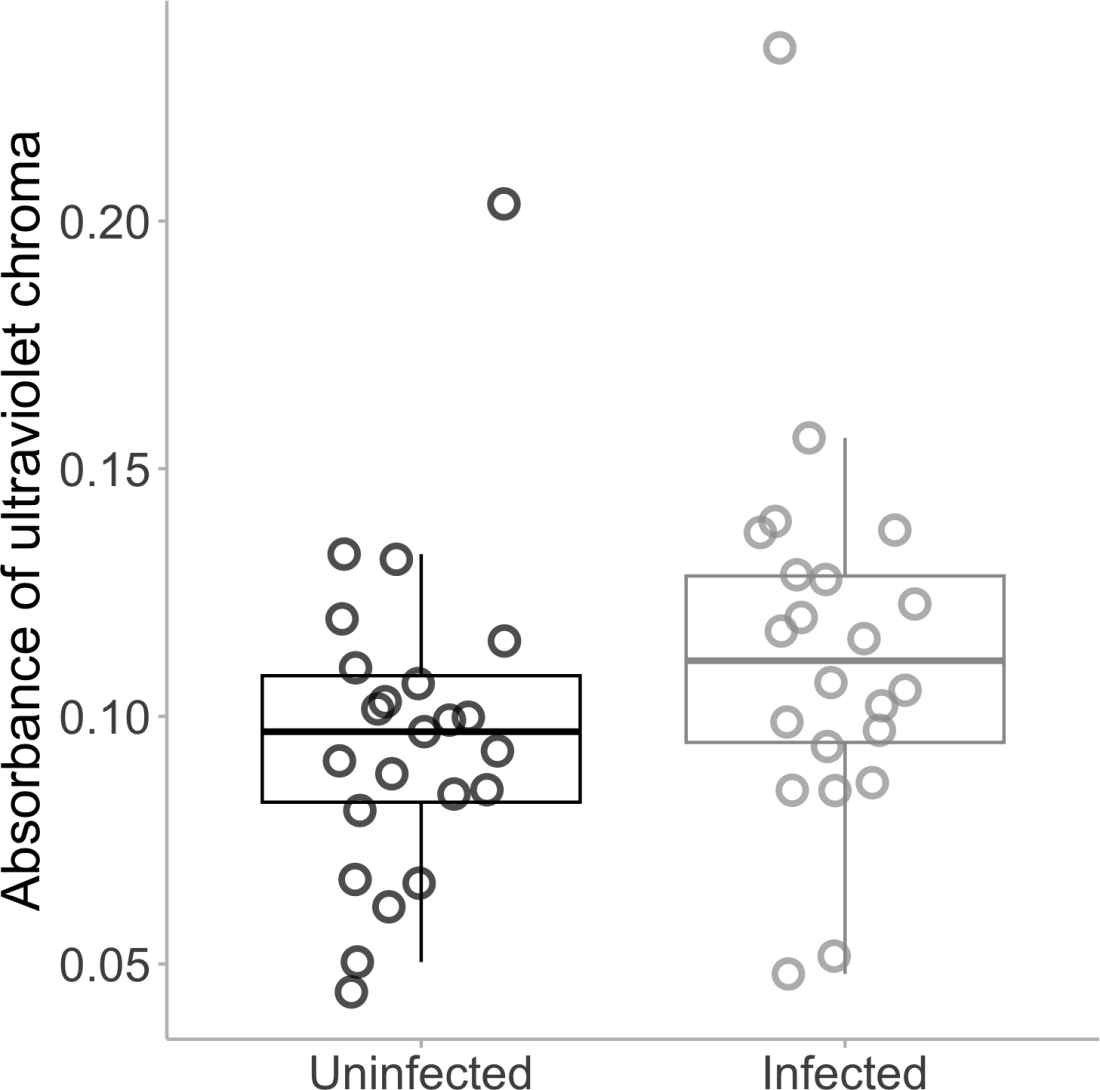
The effect of infection on ultraviolet (UV) chroma in the male throat in the field. There was a significant effect of infection status on the absorbance of UV chroma, where infected frogs had more UV chroma in their throat skin, as indicated by higher absorbance. Each point represents a single individual (23 animals were uninfected at the time of sampling, and 22 animals were infected with *B. dendrobatidis*). These single points represent an average of three colour readings per frog. The dark middle lines within the box plot represent the median value, the boxes around the median represent the interquartile range and the whiskers represent 95% confidence intervals.

We conducted a similarly designed field study to understand if breeding coloration changes with infection also occurred in the wild. We tested the throat coloration and disease status in 45 male frogs from two sites at five sampling times during their natural breeding season. Forty-nine per cent (22/45) of frogs were infected with *B. dendrobatidis* at the time that their throat coloration measurements were taken. We found increases in throat colour, where UV, instead of yellow-orange chroma, was significantly higher (14.6%, *d* = −0.43) in infected animals (LM: status, *F* = 6.334, *p* = 0.013), and increased with infection load (LM: load, *F* = 5.127, *p* = 0.026; log_10_(mass), *F* = 5.905, *p* = 0.018; electronic supplementary material, table S1; figure 3), while brightness and yellow-orange chroma remained unchanged between infected and uninfected males (electronic supplementary material, table S1). Mean UV chroma for the uninfected animals in the field and the laboratory animals prior to *B. dendrobatidis* exposure were within one standard deviation (field: uninfected UV chroma = 0.097 ± 0.037 absorbance; laboratory: week 0 UV chroma = 0.080 ± 0.023 absorbance), indicating that coloration in the laboratory and the field was similar.

We collected call recordings from a subset of the animals in the field (*n* = 23; 13 infected, 10 uninfected). Before the animals were captured, we recorded 10 min of audio to compare the effect of infection on multiple calling parameters such as call duration, pulse rate, pulses per note and notes per call. Overall, infected and uninfected males had equivalent call capacity: there was no statistically significant effect of infection status or load on calling parameters in the field (electronic supplementary material, table S2).

### 3.2. Infection led to higher quality sperm

**Figure 4.**
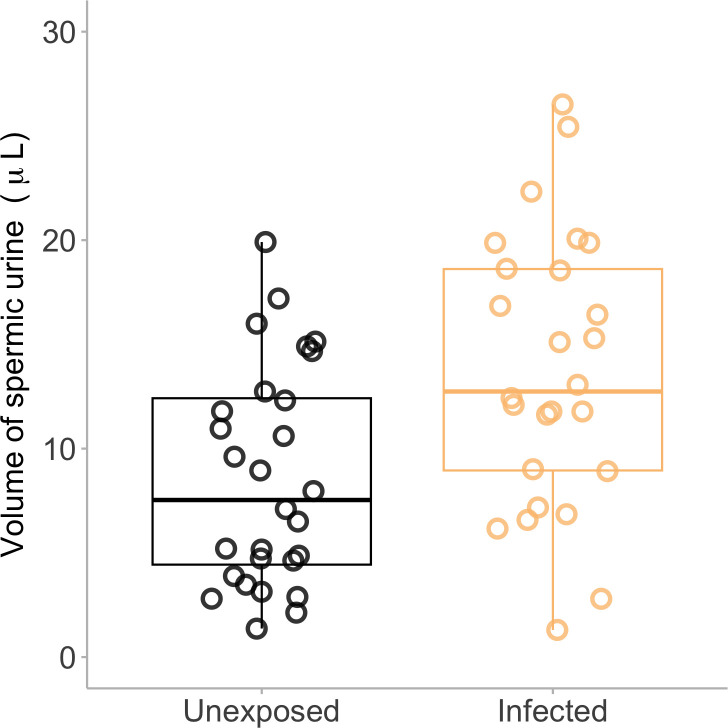
The effect of infection on spermatic urine volume during a laboratory infection experiment. There was a significant effect of infection status on spermic urine production, where infected animals produced significantly more spermic urine than unexposed animals. Each point represents a single individual (27 infected animals, and 29 animals were unexposed). The dark middle lines within the box plot represent the median value, the boxes around the median represent the interquartile range and the whiskers represent 95% confidence intervals.

In a second laboratory-controlled infection experiment, we exposed animals to *B. dendrobatidis* and collected spermic urine every two weeks (10 infected, 10 unexposed at each collection timepoint, unique animals sampled at each collection, e.g. no repeated measures) to investigate sperm quantity and quality. By sampling over the course of infection, we were able to monitor changes in sperm phenotype with disease progression. For sperm quantity, we found no statistically significant effect of infection on sperm concentration or total number of sperm cells, but there was a significant effect on the volume produced: individuals that were infected with *B. dendrobatidis* produced 65.1% more spermic urine than unexposed males (12.2 ± 2.1 ml infected; 7.4 ± 2.2 ml unexposed spermic urine; *d* = 0.67; LME, treatment, χ^2^ = 5.713, *p* = 0.017; electronic supplementary material, table S3; figure 4).

**Figure 5.**
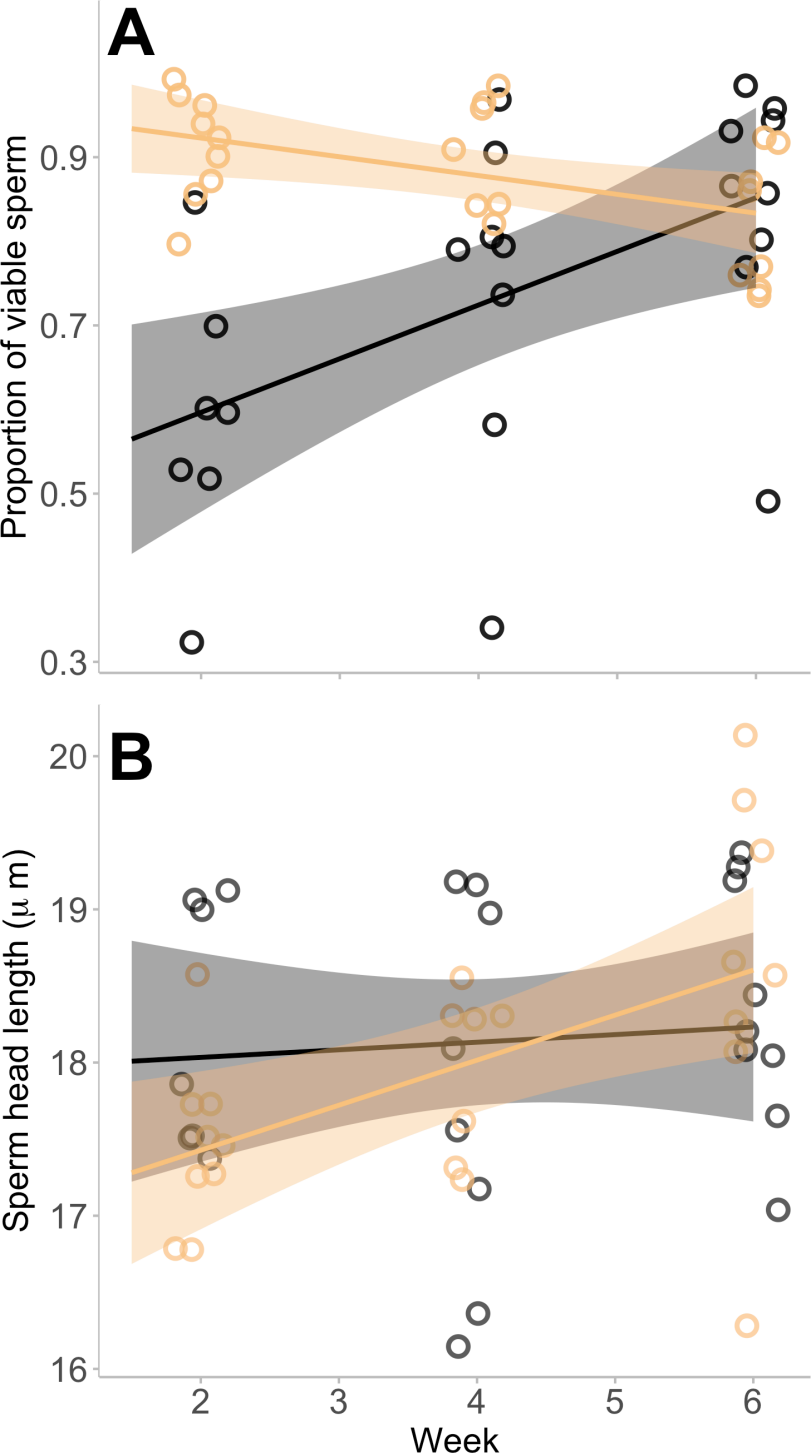
The effect of infection on sperm quality measured fortnightly over the six week experiment. (A) The proportion of viable sperm and (B) the length of the sperm head (mm). Each point represents a single individual. For (A), only one sample was taken for each individual. For (B), the single point for each individual is an average of the 20 individual sperm measured. The lines are the smoothed conditional means for each of the unexposed animals and the infected animals. The shading around the lines represents 95% confidence intervals.

In sperm quality, over the entire six week infection experiment, infected animals had an 11.9% higher proportion of viable sperm cells (cells determined to be alive via eosin–nigrosin staining technique) than unexposed animals (88.6% median viability of infected animals (10.7% interquartile range); 79.2% median viability of unexposed animals (28.3% interquartile range); *d* = 0.98). The difference between the viability of sperm cells was largest at the first sampling period, 2 weeks after inoculation, where infected animals had a 54.8% higher proportion of viable sperm compared with unexposed animals. By week 6, there was no difference in the proportion of viable sperm cells between infected and unexposed groups (GLM: treatment, χ^2^ = 11.867, *p* < 0.001; treatment × week, χ^2^ = 16.404, *p* < 0.001; electronic supplementary material, table S3; figure 5A). While sperm cell viability decreased over time in infected animals (GLM: week, χ^2^ = 11.867, *p* < 0.001; treatment × week, χ^2^ = 16.404, *p* < 0.001; electronic supplementary material, table S3; figure 5A), there was no direct effect of infection load on sperm viability (electronic supplementary material, table S3).

The morphology of the sperm was affected by infection and the time with infection. At the first sampling time post-inoculation, sperm head length was 4.4% shorter in infected animals (sperm head length of infected animals 17.6 ± 0.6 mm; 18.2 ± 0.8 mm in unexposed animals), but by week 6 post-inoculation, the sperm head length in infected animals became 1.4% larger than the sperm from unexposed frogs (sperm head length of infected animals 18.6 ± 1.2 mm; 18.4 ± 0.8 mm in unexposed animals), while control sperm head morphology remained similar in size over time (LME: treatment × week, χ^2^ = 5.277, *p* = 0.022; electronic supplementary material, table S3; figure 5B). There was no direct effect of infection load on sperm head length or of infection on sperm tail length (electronic supplementary material, table S3). There was no statistically significant effect of infection on sperm motility (electronic supplementary material, table S3).

### 3.3. Infected males bred more in the field than uninfected males

**Figure 6.**
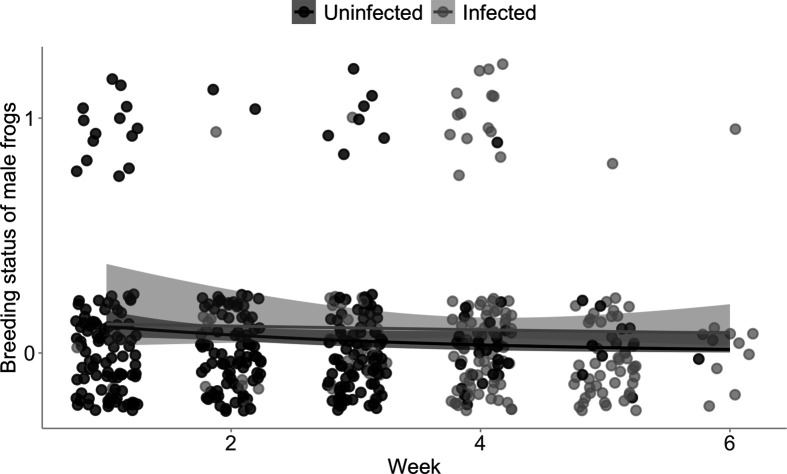
The breeding status of male frogs at every point of capture over the breeding season. Breeding status of a male at the point of capture was determined through genetic analysis of egg masses laid over the breeding season, and through capturing males in active amplexus with a female. The points on the figure represent individual capture events of males each week of the survey: 451 capture events of 179 individual male *L. v. alpina*, with 38 male capture events where the male breeding status = 1 (spawned or found in amplexus). Upon each capture, male frogs were sampled for disease status. The lines represent the smoothed conditional means using a binomial distribution and the shading around the lines indicates 95% confidence intervals.

We collected the breeding status and activity of males in the field over six weeks of the *L. v. alpina* breeding season. The egg masses produced during the breeding season had a median of 90.5 (first and third quartile: 58.5−137.5) eggs. Males that were infected with *B. dendrobatidis* were overall 31.4% more likely to successfully spawn (either produced a viable egg mass as determined via genetic parentage analysis or were found in amplexus with a female; see electronic supplementary material, text S1) than uninfected males (GLME: disease status, χ^2^ = 3.022, *p* = 0.082; electronic supplementary material, table S4; figure 6). Furthermore, time across the breeding season impacted breeding status with more males breeding earlier in the season (GLME: week, χ^2^ = 2.870, *p* = 0.090; electronic supplementary material, table S4; figure 6). Infection dynamics changed throughout the breeding season, with more infections occurring later in the breeding season. Over the whole season, fewer males were infected than uninfected (36.9% infected male frogs over the whole season).

## 4. Discussion

We found that male frogs increased their breeding effort and success through increased vocal sac coloration, sperm quality, and mating than uninfected males. Here, we have used a holistic approach to understanding the impacts of infection on breeding by assessing the impacts of infection on both primary and secondary sexual characteristics, as well as on direct breeding output. It is well understood that primary and secondary sexual characteristics can influence breeding success [42–44]; however, the breeding output (e.g. instances of successful breeding) is not often demonstrated. And while we did not directly test that these primary and secondary characteristics lead to increased breeding output, we show several different lines of evidence that *B. dendrobatidis* infection increases breeding effort and success in *L. v. alpina*.

We found that breeding display increased through yellow-orange coloration in the laboratory and UV coloration in the field in the vocal sacs of male frogs when the animals were infected with *B. dendrobatidis*. The increase in coloration was also directly associated with infection load. Colour and display of the vocal sac influence mate choice in other nocturnal amphibian species, where female frogs preferred males with more intense vocal sacs [43,45], and those males sire more offspring [46]. UV chroma is a common signal for nocturnal vertebrate animals [47] and is a known sexual signal in reptiles [48,49] and amphibians such as *Rana arvalis* [50]. While not all nocturnal animals can see UV chroma, many frogs do, including species phylogenetically close to *L. v. alpina* [51]. Parasite infection can change mating displays, for example male frogs with ectoparasites have increased mating colorations, and therefore their attractiveness to females led to reproductive success [46]. The effect size of infection on throat coloration was medium–large (Cohen’s *d* = 0.73). However, this does not prove biological relevance and there is relatively little known about levels of throat coloration in frogs that influence attractiveness. For example, many studies that test coloration do not report the effect sizes [50,52–54]. But, because we found an effect only on throat coloration while there was no change (brightness, UV, yellow-orange) across the other skin areas (dorsum or venter), this indicates that the colour change is not due to general skin pathology caused by infection. Instead, this result supports that infected male *L. v. alpina* are specifically increasing their sexual display and display effort. We do not know exactly how our spectrophotometer spectrograph colour results relate to the number of pigments in the skin, but we do know that UV and yellow chroma are structural pigments and they are costly to produce and maintain [55]. Understanding what cells are contributing to these observed colour changes is important future work.

Although our laboratory infection results showed an increase in yellow-orange coloration in the throat, they also showed a decrease in UV coloration. In the laboratory study, all animals including the unexposed control frogs decreased UV coloration in the throat over time, which could indicate that laboratory conditions do not mimic field conditions and the maintenance of UV chroma and thus not invested in [55]. Environmental factors like temperature or nutrition can affect UV coloration [56,57]; therefore, instead of investing in costly UV chroma under environmental conditions that do not support high UV chroma in the skin, the decrease in UV chroma in the infected animals could compensate for the increase in yellow-orange chroma. Further research is required to understand drivers of investing in particular pigments. Regardless, under both field and laboratory scenarios signalling chroma within the vocal sacs increased with infection, whether it be UV chroma in the field or yellow-orange chroma in the laboratory.

We found that infected males increased their sperm quality when infected with *B. dendrobatidis*. The volume of spermic urine was much higher in infected animals throughout the laboratory experiment, while the sperm concentration and total sperm numbers remained statistically equivalent between infected and unexposed animals. An increased volume of spermatic urine might give males a higher chance of fertilizing eggs by allowing the sperm to be distributed across a wider surface area and contact more eggs [58]. The non-sperm components of spermic urine enhance sperm survival in the extra-organismal environment, contributing to higher motility and viability across the aquatic spawning site [59]. These non-sperm components optimize the osmolality and pH of the urine to facilitate spermic movement and preservation [59]. Because *L. v. alpina* externally fertilizes in ponds, this increase in spermic urine might protect the sperm long enough for them to find and penetrate the eggs. Because sperm samples were small per individual, we were unable to measure total protein concentration along with the other measurements we took, but it is unlikely that infection affected hydration of the spermic urine. While chytridiomycosis does affect electrolytes in the blood, the hydration status and many blood parameters remain unaffected by chytridiomycosis and *B. dendrobatidis* infection [60], especially at sublethal levels of disease, like the samples taken in this experiment.

Infected male frogs had higher sperm viability early in infection. Sperm cell viability is an important indicator of fertilization potential [61,62], and is a proxy for sperm quality. The peak in sperm viability in week 2 of the infection experiments supports the terminal investment hypothesis. Yet, perhaps, a sustained increase in sperm viability effort throughout the six week experiment was too costly, and, as the health of frogs became compromised, sperm quality returned to baseline.

Sperm cell head length was affected by infection status but had the opposite response of sperm viability and increased over time. Early in the experiment, infected animals had shorter sperm head length but this increased as infection progressed. Sperm head length is an important trait for externally fertilized species with thick egg capsules because sperm with longer heads are better at penetrating the egg [63]. The delayed increase in sperm cell head length over the infection period might reflect the spermatogenesis cycle of frogs; the whole cycle can take four weeks [64], and we would expect a lag between exposure and change in sperm morphology.

Crucially, we found that infected males were participating in 31% more breeding events in the field than uninfected frogs. There are only a few studies that directly link the impacts of infection or another stressor to reproductive success, such as frogs infected with ranavirus breed non-randomly, where infected animals choose different mates compared with uninfected animals [65]. Here, infected males bred more readily throughout the season. Higher breeding effort and success induced by chytridiomycosis infection are supported across the experiments in this study, which assessed both secondary and primary sexual characteristics in male *L. v. alpina*. Unfortunately, we did not capture enough females within the population to analyse how the disease affected their reproductive success. Future research should aim to understand how disease impacts reproductive effort and breeding success in females.

Understanding the impacts of disease on multiple aspects of reproduction is imperative so that we can identify which mechanisms of reproduction are impacted by disease, and use that understanding to effectively conserve populations and ensure that our management strategies are appropriate. Reproduction is key to the recovery trajectory of a species; however, managing the impacts of disease on reproductive success is often overlooked in favour of mitigating disease-induced mortality through manipulation of environmental factors or immunity. Here, we provide clear key evidence for a functional reproductive change in response to *B. dendrobatidis* infection that explains how species can survive with ongoing high mortality. This builds on indirect evidence for increased reproduction, based on increased calling and testes parameters [9–11,22]. Previous research into the impacts of disease and reproduction also highlights the potential that increased reproductive effort evolves with disease endemism [10].

In this study, we explored multiple lines of evidence across several male sexual traits that can be affected by disease. We found clear evidence of terminal investment in *L. v. alpina*, where reproductive effort increases when *L. v. alpina* are infected with the devastating disease chytridiomycosis, and this ultimately leads to increased breeding success. Elucidating how some frogs are persisting despite devastating disease will direct and optimize management efforts. To reverse population declines, we need management techniques targeted to species’ life history and their specific response to endemic disease [8]. The key conservation and management practices for species that persist due to their prioritiszing breeding like *L. v. alpina* should focus on, in the short term, supporting recruitment and expanding breeding habitat. For example, it has been suggested that conservation efforts involve habitat modification to reduce transmission, like increased salinity or increased microhabitat temperature [66], and while this might be a successful approach for some species, it could reduce breeding capacity in others. Strategies targeting recruitment, which include protecting breeding habitats from desiccation or increasing pond hydroperiod (e.g. via dams) in habitats that frogs already use, can help ensure that recruitment remains high. Protecting and increasing breeding habitat will help populations persist, but fecundity compensation likely has its limits; therefore, controlling other threats is also critical, especially in a changing environment where drought can severely impact breeding potential. Importantly, by protecting breeding habitat, managers could be giving the species more time to develop stable, long-term strategies like increased resistance to the pathogen. We urge managers to integrate species life history and natural responses to disease with conservation efforts to mitigate disease-threatening processes. This approach to amphibian conservation presents a feasible and complementary alternative to the challenge of directly combatting this pandemic disease.

## Data Availability

All data used in this experiment have been uploaded to Dryad [67]. Electronic supplementary material is available online [68].
